# Low-diluted *Phenacetinum* disrupted the melanoma cancer cell migration

**DOI:** 10.1038/s41598-019-45578-1

**Published:** 2019-06-24

**Authors:** Camille Fuselier, Christine Terryn, Alexandre Berquand, Jean-Marc Crowet, Arnaud Bonnomet, Michael Molinari, Manuel Dauchez, Laurent Martiny, Christophe Schneider

**Affiliations:** 1 CNRS UMR7369 MEDyC, University of Reims Champagne-Ardenne, Reims, France; 2 Plateform PICT, University of Reims Champagne-Ardenne, Reims, France; 3 LRN EA 4682, University of Reims Champagne-Ardenne, Reims, France

**Keywords:** Cell migration, Melanoma

## Abstract

Dynamic and reciprocal interactions generated by the communication between tumor cells and their matrix microenvironment, play a major role in the progression of a tumor. Indeed, the adhesion of specific sites to matrix components, associated with the repeated and coordinated formation of membrane protrusions, allow tumor cells to move along a determined pathway. Our study analyzed the mechanism of action of low-diluted *Phenacetinum* on murine cutaneous melanoma process in a fibronectin matrix environment. We demonstrated a reduction of dispersed cell migration, early and for as long as 24 h, by altering the formation of cell protrusions. Moreover, low-diluted *Phenacetinum* decreased cell stiffness highly on peripheral areas, due to a disruption of actin filaments located just under the plasma membrane. Finally, it modified the structure of the plasma membrane by accumulating large ordered lipid domains and disrupted B16 cell migration by a likely shift in the balance between ordered and disordered lipid phases. Whereas the correlation between the excess of lipid raft and cytoskeleton disrupting is not as yet established, it is clear that low-diluted *Phenacetinum* acts on the actin cytoskeleton organization, as confirmed by a decrease of cell stiffness affecting ultimately the establishment of an effective migration process.

## Introduction

Directional motility is a physiological cellular process which is essential for embryogenesis, immune response, tissue repair, and organ formation. Cell types and environment influence the mode of migration, which can be individual (amoeboid or mesenchymal modes) or collective (cluster or sheet modes). Briefly, cell motility is characterized by a succession of steps including initiation of cell polarity, formation of protrusions at the leading edge, establishment of new adhesion sites, and rear tail retraction. This cycle dependent on multiple intracellular regulatory mechanisms involving connections with plasma cell membrane, can be controlled by extracellular matrix properties^1,2^. However, aberrant migration can also promote diseases such as cancer and metastatic spread. Indeed, during cancer progression, the migration of uncontrolled and isolated cells can cover local distances, disseminate in blood circulation, and develop metastasis in secondary organs^3,4^. Among all cancers, the rate of cutaneous melanoma has increased continually as 132,000 people are affected each year (World Health Organization). Moreover, the number of death cases has increased significantly for forty years (INCA, Institut National du Cancer, Oct 2013) especially when persistent in skin, lungs, lymph nodes, intestinal organs and brain. So, while primary cutaneous melanoma are mostly treated with a curative wide local excision^5^, in the case of a metastatic dissemination diagnosis, other therapeutic strategies have to be initiated. Among them chemotherapy (vemurafenib or dacarbazine), immunotherapy which represents a promising way of treatment targeting immune check points (PD1, Programmed Cell Death; CTLA, Cytotoxic T-Lymphocyte-Associated Protein), targeted therapy using inhibitors of B-RAF or cell therapy meant to boost T lymphocytes activity and radiotherapy (localized ionizing radiation), are used alone or in combination so as to increase their efficacy^6^. However these treatments are generally considered ineffective for metastatic melanoma leading frequently to the development of chronic side effects, toxicity in patients and sometimes to therapeutic failures^5,6^. Therefore, the use of complementary and alternative medicines (CAM) as integrative therapy by cancer patients, in parallel with anticancer treatments prescribed by oncologists, has increased considerably over the past 30 years^7^. Homeopathy is part of these CAM. Developed in the 18^th^ century by German physician Samuel Hahnemann, it is based on two principles: the Law of Similars (“*similia similibus curantur*; let likes be cured by likes”) and Individualisation, and it makes use of a specific form of remedy preparation, the stepwise dilution and potentisation^8–10^. The effect of homeopathy has already been recognized, especially for improving the cancer patients’ quality of life^11,12^, and many studies have only begun to demonstrate the effect of some homeopathic dilution in cancer cell lines, *in vitro*. For instance, *Lycopodium clavatum* 5 Centesimal Hahnemannienne (CH) and 15CH have the capacities to induce apoptosis in HeLa cancer cells^13^, and the “Canova method” composed of several homeopathic dilutions, can stimulate the immune system by activating macrophages^14^. Furthermore, a recent study shows that *Ruta graveolens* 30CH is able to decrease cell viability and cell migration, by increasing apoptosis of the human colon cancer^15^. Considering all these facts and despite a context that tends to question the existence of any effect related to homeopathic treatments, we have decided to evaluate the impact of low homeopathic dilutions of phenacetine *in vitro* on melanoma cell lines. The chemical basis of this homeopathic dilution is phenacetine, an aromatic organic compound known as a drug with analgesic and anti-pyretic properties, comparable to paracetamol and produced in the United States in the 1920s (IARC 1977, FDA 1999). Until 1983, phenacetine was used over-the-counter in remedies for pain and fever and also in rheumatoid arthritis, but the established presence of carcinogenicity in renal pelvis and urinary bladder caused its withdrawal from the market^16^. However, despite these harmful effects, some studies have demonstrated that the use of a substance potentially toxic yet highly diluted (such as cadmium and arsenic), can produce an effective reduction of their usual toxic aspect *in vitro*/*in vivo* and increase beneficial application^17–19^. Based on this knowledge, the present study will describe for the first time the effects of low-diluted *Phenacetinum* (*Phenacetinum* 4CH – 1 × 10^−8 ^_M_), on cancer cell migration *in vitro* for murine cutaneous melanoma cell lines. Indeed, the combination of different original methodologies makes it possible for *Phenacetinum* 4CH to disrupt lipid organization at the plasma membrane, affecting underlying cytoskeleton performance and thereby, dispersed cell migration.

## Results

### *Phenacetinum* 4CH decreases 2-dimensional (2D) and 3-dimensional (3D) dispersed B16 cells distance and velocity migration

**Figure 1:**
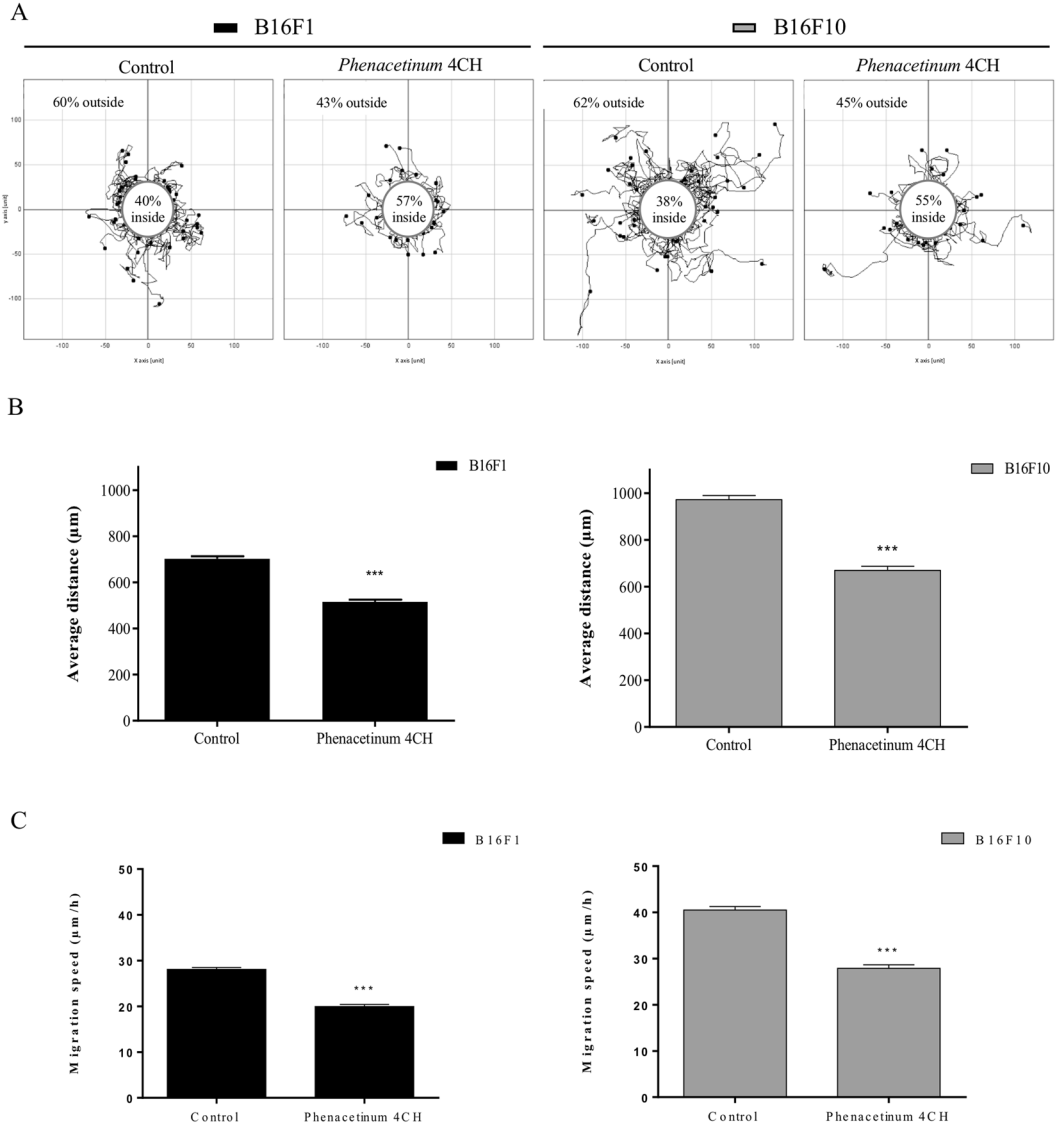
Effect of *Phenacetinum* 4CH on dispersed B16 cells distance and velocity 2D migration. For 2D dispersed migration, B16 cells were seeded on fibronectin [1.2 μg/cm^2^] and tracked for 24 h using *Manual Tracking* plugin. (**A**) Data obtained were analyzed by chemotaxis plugin (trajectories of 60 cells per image) on ImageJ software, and quantification of average distance traveled in 24 h was expressed in μm, n = 8 for B16F1 and n = 3 for B161F0, ***p < 0,001 (**B**).

**Figure 2:**
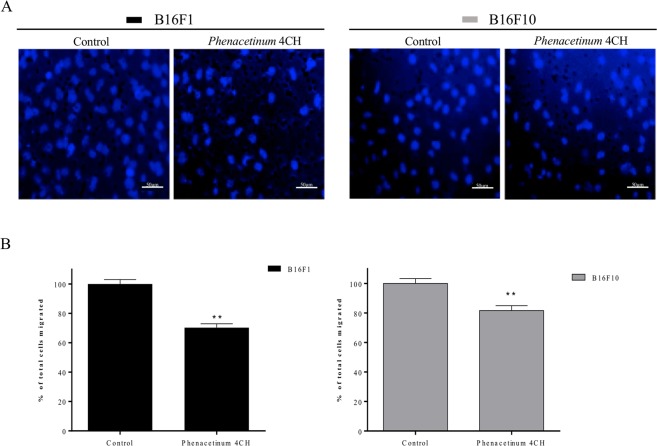
Effect of *Phenacetinum* 4CH on dispersed B16 cells 3D migration. For 3D cell migration, B16 cells were seeded on Boyden chamber coated with fibronectin [1.2 μg/cm^2^] and allowed to migrate for 6 h. Percentage of nuclei (under the polycarbonate membrane) was counted by ImageJ software, n = 4, (scale are, 50 μm). (**C**) Data are expressed as means ± SEM.

Figure 1 depicts the 24 h effect of *Phenacetinum* 4CH, on 2D dispersed B16 cells migration on fibronectin coating. Pictures in Fig. 1A, represent 60 trajectory profiles take randomly and blindly depending on the following conditions. The initial position of each cell was set at the origin (0,0) of coordinates, and the white circles in the center were determined to have about 2/3 control of the B16 cells outwards. In these conditions, representative tracks showed that among the 60% of cells outside the circle in control situation, only 43 to 45% were out when they were treated with *Phenacetinum* 4CH. Then, the diminution between the control cells and the treated cells outside circles was at 28 and 27.5% for B16F1 and B16F10 cells respectively. Supplementary information on Fig. 1B, obtained by tracking the total migratory paths on 24 h of random cells, allowed us to determine that B16F1 control cells migrated on average at 694 ± 11 μm for 24 h and B16F10 cells at 972 ± 18 μm. Under *Phenacetinum* 4CH treatment, the migratory capacities of B16 cells were significantly reduced by 27% at 510 ± 9 μm for B16F1 cells, and by 31% at 670 ± 18 μm for B16F10 cells. Moreover, this diminution was apparent after 2 h, and sustainable up over 24 h (data not shown). In addition, analysis of the total migratory speed of random cells during 24 h, enabled to identify that B16F1 control cells migrated on average at 28.1 ± 0.4 μm/h and B16F10 cells at 40.5 ± 0,4 μm/h (Fig. 1C). Under *Phenacetinum* 4CH treatment, the migration capabilities were significantly reduced by 29% at 20 ± 0.8 μm/h for B16F1 cells, and by 31% at 27.9 ± 0.8 μm/h for B16F10 cells (Fig. 1C). These results confirm that *Phenacetinum* 4CH decreased the distances of the cell migration by a decrease of the B16 cell migration speeds in a fibronectin context. Furthermore, we confirmed that the effect of *Phenacetinum* 4CH specifically acts on cell migration mechanism since we did not find any significant alteration in the cellular viability after 24 h of treatment compared to control cells (Supplementary Fig. S1). In a complementary way, we tested the effect of *Phenacetinum* 4CH on murine normal cells (Murine Embryonic Fibroblasts) to determine its specific action on cancer cell line. Indeed, there is no difference of migration distances during 24 h between control MEFs cells (215 ± 10 μm) and cells treated with *Phenacetinum* 4CH (217 ± 9 μm) (Supplementary Fig. S2A). In the same way, the migration velocity was not changed between control MEFs cells (21.5 ± 1 μm/h) and treated MEFs cells (21.7 ± 0.9 μm/h) (Supplementary Fig. S2B). Finally, we confirmed the effect of *Phenacetinum* 4CH in 3D migration using Boyden chamber, coated with fibronectin (Fig. 2). Indeed, our homeopathic dilution reduced significantly B16 cells migration after 6 h treatment of 29% for B16F1 cells and 18% for B16F10 cells, as shown on the pictures and histograms quantification (Fig. 2A,B). These results allow us to grant *Phenacetinum* 4CH an inhibitory role on dispersed B16 cells migration in a fibronectin context.

### *Phenacetinum* 4CH decreases B16 cell polarity and living cells stiffness

**Figure 3:**
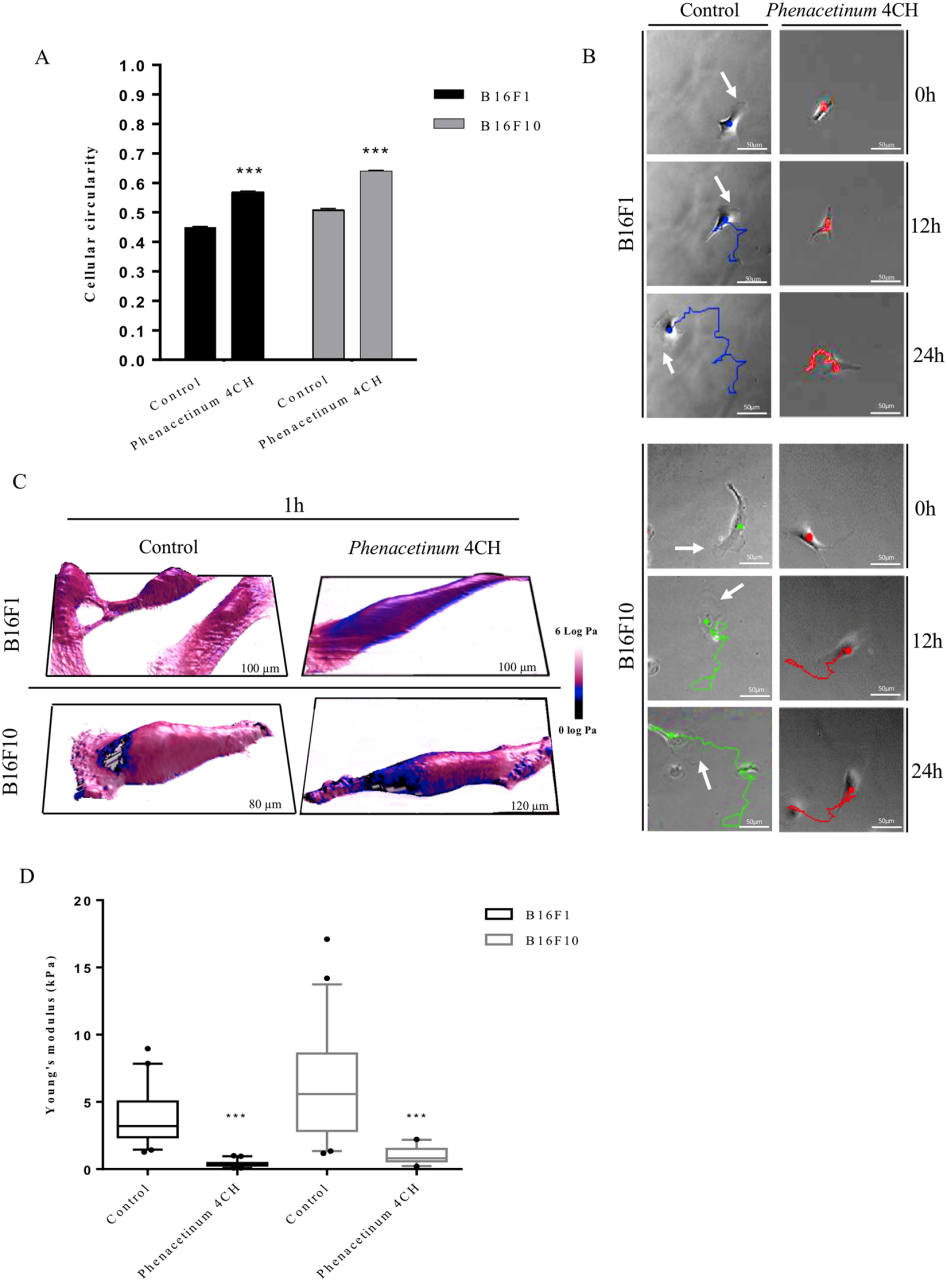
Influence of *Phenacetinum* 4CH on cell circularity and cell stiffness of living individual B16 cells. The average cell circularity (from 0 to 1 in the sense of circularity) of these cells was studied on same cells with ImageJ, n = 4 for B16F1 and B16F10 cells, ***p < 0,001 (**A**) and the typical profile of trajectories of these cells (scale bar, 50 μm), treated or not during 24 h, is illustrated in (**B**). After 1 h of treatment, cell stiffness of dispersed living B16 cells, seeded on fibronectin [1.2 μg/cm^2^], were studied and their elastic properties (Pa). Topographic images are represented in 3D while the color code is linked to the cell Young’s modulus values (log Pa unit) according to the experimental condition. Both are representative of the cell behavior for each condition. (**C**) The average values of Young’s Modulus on perinuclear areas are represented in box and whiskers, n = 6 for B16F1 and B16F10 cells, ***p < 0,001. (**D**) Data are expressed as means ± SEM.

Efficacy of cell migration is caused by a succession of membrane protrusions allowing the development of cell polarity. To investigate morphological changes of cells induced by *Phenacetinum* 4CH, their circularities have been studied. The histogram presented in Fig. 3A demonstrates, than on average over 24 h, that control B16 cells showed a balance between circular (1) and polarized (0) phenotype with values at 0.45 ± 0.002 for B16F1 cells and 0.51 ± 0.002 for B16F10 cells. In control, these cells spread and advanced along a determined and polarized trajectory from pathways over 24 h, due to flat protrusions at the leading edge apparent at 0 h, 12 h and 24 h (white arrows on blue and green pathways, Fig. 3B). On the contrary, treated cells exhibited a more circular phenotype (Fig. 3A), of 26% for B16F1 cells with values of cell circularity at 0.57 ± 0.002 and 25% for B16F10 cells with values at 0.64 ± 0.002. Indeed, treated cells revealed reduced and randomized trajectories observable after 12 h with B16F1 cells, and mainly 24 h with two cell types (red pathways, Fig. 3B). The impact of *Phenacetinum* 4CH on migration distance and formation of cell protrusions prompted us to study the mechanical properties of cells. Determination of cell stiffness was conducted with Atomic Force Microscopy (AFM) by Peak Force Quantitative Nanomechanical Mapping measurements (PFQNM mode). Indeed, AFM nanomechanical measurements combine imaging and indentation spectroscopy to map the spatial distribution of cell mechanical properties, which in turn reflects the structure and function of the underlying cytoskeleton^20^. Analyzes were made on perinuclear areas, to get rid of the nucleus and membrane extensions which will lead to artefacts in the mechanical measurements. For the different conditions, Fig. 3C shows the topography of the cells in 3D while the color code is linked to the values of the reduced Young’s modulus (elasticity modulus). After 1 h, B16 treated cells showed an important decrease of Young modulus (characterized by blue color instead of the pink one for the non-treated cells, Fig. 3C). *Phenacetinum* 4CH caused a decrease of cell stiffness by factor 9.5 (3.8 ± 1.9 kPa to 0.4 ± 0.2 kPa) for B16F1 cells, and by factor 5.8 (5.9 ± 3.7 kPa to 1.0 ± 0.6 kPa) for B16F10 cells (Fig. 3D). In addition, *Phenacetinum* 4CH did not alter MEFs cell stiffness (Young modulus value at 8.4 kPa ± 0.8 for control fibroblasts and 7.7 kPa ± 0.9 for treated fibroblasts) which implies that it has no effect on the actin cytoskeleton integrity (Supplementary Fig. S3A,B). As a conclusion, these results indicate that *Phenacetinum* 4CH is able to specifically decrease B16 cell stiffness, disturbs the cell polarity, and quickly influences the persistence of the trajectories followed by cells.

### *Phenacetinum* 4CH disrupts actin filaments of living B16 cells

**Figure 4:**
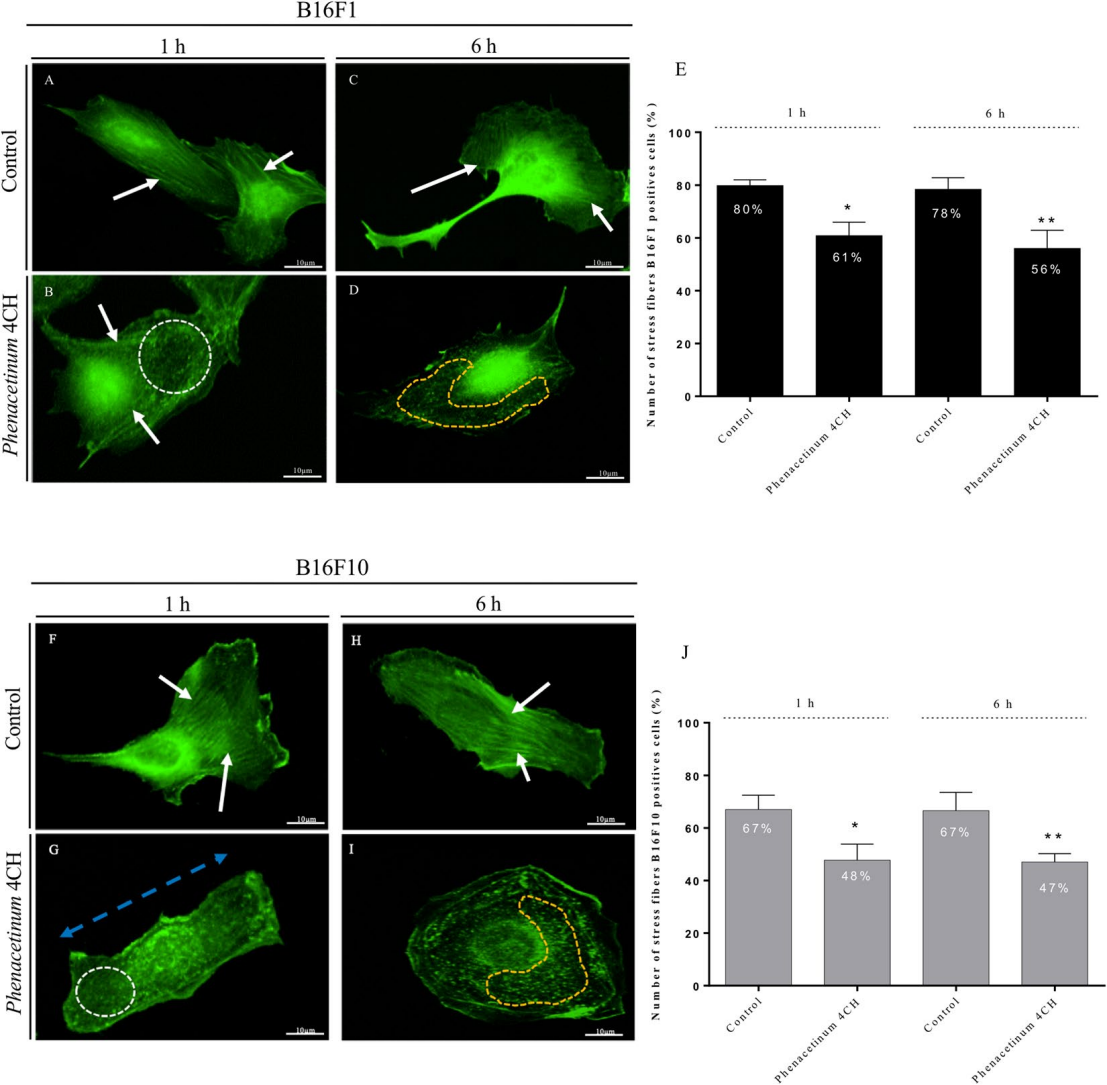
Behavior of *Phenacetinum* 4CH on actin cytoskeleton organization of living individual B16 cells. B16 cells were seeded on fibronectin [1.2 μg/cm^2^] and treated for 1 h and 6 h with *Phenacetinum* 4CH. Cells were fixed with glutaraldehyde, permeabilized with TritonX-100, and stained with Phalloïdin-Alexa Fluor 488 (actin filament in green). Samples were visualized using epifluorescence microscope, objective 20× (scale bar, 10 μm). The white arrows show actin stress fibers (**A**–**C**,**F** and **H**), dotted blue arrow shows a loss of polarity (**G**), dotted white and orange circles show a disruption of cortical actin (**B**,**D**,**G** and **I**). Profile types like stress fibers (organized actin networks) was quantified for B16F1 (**E**) and B16F10 cells (**J**) in each condition and representative of presented data. They are expressed in percentage, n = 4 where 60 cells were analyzed per condition and experiment, (ANOVA *p < 0.05 **p < 0.01).

To confirm results obtained on cell stiffness, we assessed the effect of *Phenacetinum* 4CH on actin cytoskeleton by immunofluorescence assay (Fig. 4). After 1 h, control cells were polarized with longer aligned and parallel stress fibers (white arrows, Fig. 4A,F). Treated cells showed a few stress fibers (white arrows, Fig. 4B) with cortical actin beginning to disrupt (white dotted circles, Fig. 4B,G) and sometimes lose their sense of polarity (dotted blue arrow, Fig. 4G). After 6 h of treatment, control cells were still polarized with a leading edge, retraction tail and stress fibers (white arrows, Fig. 4C,H). Moreover, in contact with *Phenacetinum* 4CH for 6 h, cells adopted a more circular phenotype along with an important disruption of cortical actin (orange dotted circle, Fig. 4D,I). Finally, the control cells exhibited mainly an actin cytoskeleton network with stress fibers highly organized (white arrows) and on the contrary, treated cells with *Phenacetinum* 4CH induced an important disintegration of actin cytoskeleton network. After 1 h or 6 h, 80% of control B16F1 cells showed a stress fiber network (Fig. 4E). B16F10 control cells exhibit 67% of stress fibers cytoskeleton (Fig. 4J). So, in a general way, the majority of the B16 control cells population maintain an organized actin network over time. Ultimately and consistently with previous images, after 1 h or 6 h of treatment with *Phenacetinum* 4CH the quantity of cells positives for cell fibers was decreased of 30% for B16F1 cells (Fig. 4E) and of 25% for B16F10 cells (Fig. 4J). These results demonstrate that *Phenacetinum* 4CH causes an increase of the actin network destruction and disorganization of B16 cells on fibronectin, while this being known to play a crucial role in cell migration.

### Phenacetin molecules interact with membrane by way of molecular dynamic (MD) simulation

**Figure 5:**
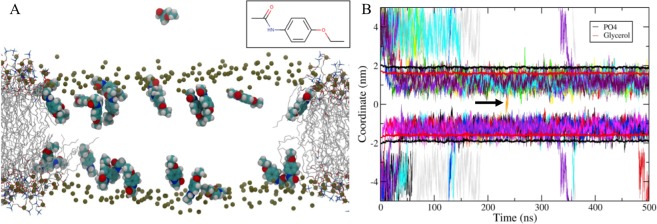
Molecular dynamics simulations of 25 phenacetins in presence of a POPC bilayer, and hydrogen bonds formed by phenacetins. Representative snapshot of the system with phosphorus (tin color) and phenacetins depicted using the van der Waals representation and the lipids with a line representation. (**A**) The phenacetin structure is presented in the inlet panel. Z coordinates of the phenacetin’s center of mass. (**B**) Black and red curves represent the phosphorus and glycerol positions and black arrow demonstrates orange curve.

Our data suggest that phenacetin action could be the result of an interaction with plasma membranes. In order to explore the localization of phenacetin at the membrane, we developed an original approach using molecular dynamic simulations. MD simulations of 500 ns were performed on several lipid bilayers 1-palmitoyl-2-oleoylphosphatidylcholine (POPC), 1,2-dipalmitoylphosphatidylcholine (DPPC), and DPPC:cholesterol, of an increasing degree of order, and with a phenacetin/lipid ratio of 1/10. At the beginning of the simulation, phenacetin molecules were positioned outside the bilayer at around 1 nm of the membrane surface. As the simulation ran on, phenacetin molecules rapidly insert into close interaction with the bilayer as seen in both panels of Fig. 5A, which highlights the penetration of the phenacetin inside the hydrophobic core (the center of the mass of phenacetins was in average at 1.2 nm from the POPC membrane). Phenacetin behaved similarly with the three lipid membrane compositions. Figure 5B also shows that this molecule is able to cross the lipid bilayer (orange curve presented by black arrow) and get out of the membrane into the bulk. A more thorough examination of the position of phenacetin molecules during the trajectory (Fig. 5A) revealed that phenacetin adopted a specific orientation in the membrane with its amide group near lipid polar heads and its more deeply inserted ether group. Phenacetin had an optimum angle of around 20° relative to the bilayer normal, which decreased with the membrane order (see Supplementary Fig. S4). Phenacetin formed hydrogen bonds with lipid head groups, mainly glycerol, and was also able to form hydrogen bonds with itself (Table 1). Phenacetin can indeed form small clusters as shown in Supplementary Fig. S5 and a RDF between 2 and 3 as well.

**Table 1:** Phenacetins interactions.

	POPC	DPPC	DPPC:CHOL (90:10)
COC – CON^a^	0.12	0.06	0.08
CON – CON	3.39	2.96	4.97
CON – Phosphate	8.66	7.64	5.61
CON – Glycerol	21.37	23.77	23.30
COC – Cholesterol			0.45
CON – Cholesterol			1.00
CON – water	127.88^b^	112.94^b^	106.20^b^
COC – water	14.94	9.73	6.19

Values correspond to the percentage of the simulation time during which hydrogen bonds are observed per phenacetin.

^a^COC and CON correspond respectively to the ether and amid group of the phenacetin.

^b^Values greater than 100 means that several hydrogen bonds are formed at the same time.

### *Phenacetinum* 4CH increases lipid ordered domains of living B16 cells

**Figure 6:**
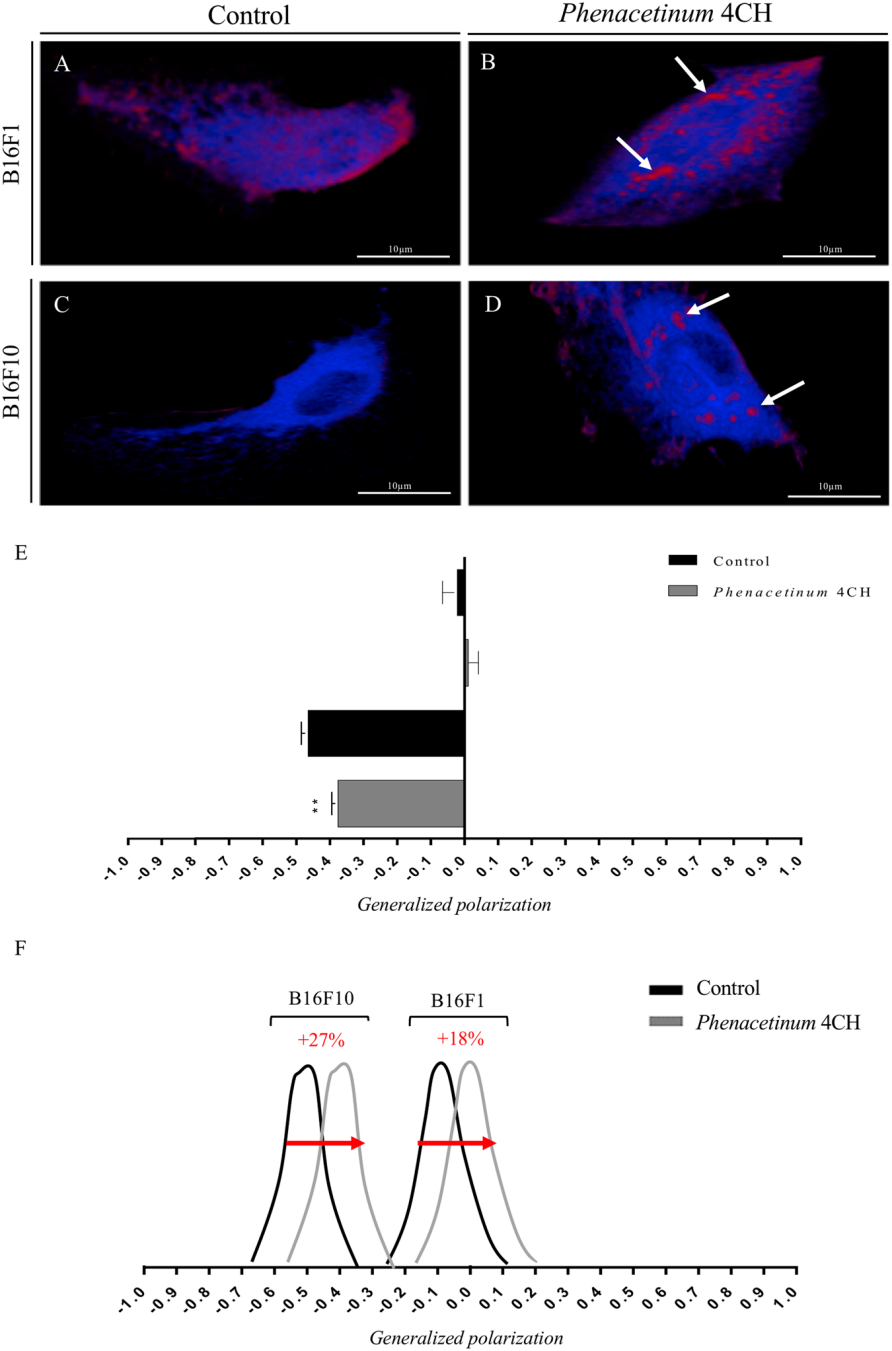
Effect of *Phenacetinum* 4CH on structural membrane of living individual B16 cells. B16 cells were seeded on fibronectin [1.2 μg/cm^2^], incubated with Laurdan (di-4-ANEPPDHQ) [5 μM] overnight at 37 °C and then treated with homeopathic drug. After 1 h, living cells were excited at 800 nm with bi-photon confocal microscope, immersion objective 60× (scale bar, 10 μm). Then, camera capture two simultaneous images with wavelengths range to 400–460 nm (liquid-ordered phase ≪lo≫, in red) and 470–530 nm (liquid-disordered phase ≪ld≫, in blue). White arrows show ordered lipid phases. (**A**–**D**) Emission intensity of pixels was introduced in generalized polarization equation which provides GP values as a measurements of membrane fluidity. Quantification of GP values are ranged from −1 (more fluid) to +1 (more rigid), n = 4 for B16F1 and B16F10 cells, *p < 0.05 (**E** and **F**). Data are expressed as means ± SEM.

In order to investigate the benefits gained from the *Phenacetinum* 4CH mechanistic effect on B16 cells and on basis previous MD information, we studied its action on the phospholipid organization contained in the plasma membrane of cells plated on fibronectin. The data presented on Fig. 6 show a different distribution of fluorescence depending on conditions after 1 h of treatment. Results indicated (Fig. 6A) homogeneity between disordered lipid phases (Ld, in blue fluorescence) and ordered lipid phases (Lo, in red fluorescence) in B16F1 control cells, unlike B16F10 cells (Fig. 6C) showing a majority of Ld phases. When cells were treated with *Phenacetinum* 4CH, we observed large and localized Lo domains (white arrows, Fig. 6B,D). In order to measure and quantify plasma membrane order, the histogram presented in Fig. 6E, indicates generalized polarization (GP) values of perinuclear areas. In control, we noticed that B16F1 had an average GP value of −0.04 ± 0.03 and B16F10 an average GP value of −0.47 ± 0.02. Homeopathic drug treatment causes an increase of GP values of 0.01 ± 0.03 and −0.37 ± 0.02 for B16F1 and B16F10 respectively. To conclude, *Phenacetinum* 4CH induces a 27% shift in B16F10 cells and 18% in B16F1 cells to increase their membrane stiffness on fibronectin and after 1 h of treatment (Fig. 6F).

## Discussion

In general, results of *in vitro* studies, animal studies and clinical trials suggest that combine homeopathy with conventional cancer care might have some beneficial effects against tumor progression and mainly on the life quality of patients suffering from cancer^11,12,21^. Interestingly, it has been observed that global health status, fatigue symptoms and other side effects did improve during cancer treatments accompanied with homeopathy^21^. Concerning cutaneous melanoma, it is well established that primary tumors are mostly treated by means of a curative local excision, but the survival rate of patients is largely reduced when metastases are diagnosed^6^. Evidence of efficacy being disputed, more study and particular preclinical study on homeopathy associated with allopathic treatments are needed aiming to reduce adverse effect. Indeed, several *in vivo* and *in vitro* preclinical studies have investigated the effect of homeopathy in different cancers. More particularly in the case of metastatic melanoma cancer, Sunila *et al*. highlighted a significant inhibition of lung metastasis with B16F10 cells by *Thuja occidentalis*, a homeopathic alcoholic extract induced in murine model^22^. This was confirmed by others using a composed homeopathic mixture, called M1 product^23^. Regarding *in vitro* axis, Ghosh *et al*. demonstrated that the homeopathic mother tincture of *Phytolacca decandra* induces apoptosis of A375 melanoma cells by activating caspases-mediated signaling and ROS generation^24^. On the other hand, no effect was observed on melanoma cells with *Zincum metallicum*^25^. Although these previous studies are encouraging, the particular action of the mechanism remains unclear.

In this study, we defined the specific conditions associated with a dispersed cell culture model to investigate *in vitro* for the first time the role of the homeopathic *Phenacetinum* 4CH dilution, on murine melanoma cell lines. To mimic the physio-pathological conditions connected with cutaneous melanoma progression, we analyzed the behavior of B16 cells grown on fibronectin matrix^26–29^. To better address the action mechanisms of *Phenacetinum* 4CH, its effect on murine normal cells as a negative control was tested. Interestingly the lack of effect of *Phenacetinum* 4CH on key parameters of MEFs cells, such as cell migration and cell stiffness, strongly confirms its specific action on cancer cells. Our data showed that under *Phenacetinum* 4CH treatment, the development of membrane protrusions was anarchic, which reduced quickly and sustainably the dispersed cell migration. Moreover, we demonstrated that this treatment induced disruption of actin filaments underlying plasma membrane. Finally, we considered that reinforced by MD simulations, *Phenacetinum* 4CH upset the lipid membrane organization in increasing Lo domains. Hence, by disturbing the membrane fluidity associated to the disruption of cytoskeleton architecture and its dynamic, *Phenacetinum* 4CH altered the progression of murine melanoma cells as observed in the 2D and 3D migration experiments.

Firstly, we report that only after 1 h of treatment, the application of *Phenacetinum* 4CH on B16 cells reversed the lipid balance of plasma membrane, increasing the level of Lo phases. Plasma membrane contained a multitude of lipids and proteins, which are specific to characteristic domains known as lipid rafts. These lipid domains essential for cell signaling, are enriched in saturated lipids (mainly sphingolipids) and in sterols (mainly cholesterol) assimilated in most cases to Lo phases^30,31^. They are characterized by an important rigidity with a higher density of packing, as opposed to Ld phases composed of unsaturated lipids and defined as fluid^30–32^. Based on this knowledge, we suggest that *Phenacetinum* 4CH may increase the rigidity of the plasma membrane, involving probably an elevation of cholesterol level. In this sense, preliminary results seem to demonstrate an increase of membrane cholesterol level, when B16 cells are treated with *Phenacetinum* 4CH (data not shown). However, as described earlier, we know that membrane fluidity of cancer cells was directly and significantly correlated with the malignant potential of these cells^33–35^. For example, Hendrich *et al*., hypothesized that low concentration of cholesterol in plasma membrane allowed cell membranes to be more easily deformable, making them highly metastatic^36^. Conversely, we can suppose that *Phenacetinum* 4CH treatment decreases the metastatic power of melanoma cells, through an increase of membrane rigidity. Moreover, MD simulations allowed us to understand better atomic interactions with model membranes, as shown recently by Fei *et al*. highlighting new molecular mechanism of resveratrol^37^. Thus, resveratrol seems to show effects opposed to cholesterol in DPPC liquid phase as it decreases membrane thickness imposed by its orientation^37^. In our view, MD simulations showed that phenacetin molecules, contained in the low homeopathic dilution assessed here, were able to get spontaneously into POPC and DPPC lipid bilayers. They could position themselves perpendicularly on the bilayer surface and form clusters occasionally. In addition, their specific orientation in the membranes allowed them to establish hydrogen bridges between glycerol groups of phenacetin molecules and hydroxyl groups of cholesterol. Also, thanks to the greatly reduced complexity of the lipid membrane structure construction, these MD simulations data allowed us to consolidate the previous results indicating that *Phenacetinum* 4CH could get into biological membranes and increase the membrane rigidity through the accumulation of Lo phases. Similarly, already reported was the fact that some flavonoids molecules like Genistein could alter membrane fluidity by accumulation in lipid rafts and induce cell apoptosis through recruitment of Fas/CD95 at the plasma membrane^32,38,39^. It therefore seems that a rise of lipid rafts may influence the cellular homeostasis. Indeed, it is widely accepted that lipid rafts are involved in many cellular processes such as signal transduction, cholesterol transport, membrane trafficking, cytoskeletal organization, motility, polarity and endocytosis^40^. Chun *et al*. attested in Hela cells that an increase of membrane cholesterol levels decreased phosphatidylinositol 4,5-biphosphate (PIP_2_) via an important activation of phospholipase C (PLC)^41^. Cholesterol- and sphingolipid-rich rafts are then able to confine PIP_2_ within the plasma membrane leads to limit its activity. However, this phospholipid is an important regulator in a variety of cellular functions, since it can be involved in calcium and PI3K/AKT signaling pathways. Moreover, its presence in the plasma membrane may allow the recruitment and activation of effectors, such as guanine nucleotide exchange factors (GEFs) or GTPases accelerating protein (GAPs) to small Rho-GTPases involved in remodeling of actin cytoskeleton and cell motility^42^.

Ultimately, composition and structure of the plasma membrane and the underlying motility machinery seem to be tightly connected through a successful coordination of many cellular processes. Using the highly sensitive and spatial resolution of AFM approach, our results on cell mechanical properties reveal that after 1 h, *Phenacetinum* 4CH induces a strong reduction of B16 cell stiffness. As demonstrated in many publications, the actin cytoskeleton integrity brings a significant contribution to an optimal cell stiffness. Indeed, the depolymerization of actin filaments by different chemicals treatments like Cytochalasin D or Latrunculin B, was accompanied by a reduction of cell stiffness^20,43,44^. In our study, the application of an indentation force around 70 nm enabled to confirm that we directly analyzed cortical actin (generally between 50 and 200 nm of depth depending of the cells)^45^. In that way, the rapid increase of cell fluidity induced by *Phenacetinum* 4CH directly reflects a perturbation of the structure and/or function of the underlying actin cytoskeleton that may result in a reduction of membrane-cytoskeleton adhesion. This assumption was supported by immunofluorescence assays, since we observed a considerable disruption of actin filaments network under *Phenacetinum* 4CH treatment. Thus, the membrane-cytoskeleton system governs cellular elastic properties, essential for cell migration^45^.

Cells contain two types of actin filaments. Stress fibers are parallel to the direction of movement and bind themselves to myosin II to provide contraction force. Conversely, cortical actin branched network is polymerized under the plasma membrane which promotes cell deformation by protrusion formation^45,46^. Among these membrane protrusions, the lamellipodia generate protrusive activity at the leading edge, involving polarity and cell motility. On the contrary, our present results demonstrated that *Phenacetinum* 4CH was able to impede the polarity of B16 cells by the development of many peripheral lamellae, which increase the cell circularity. Indeed, involvement of many proteins to generate a lamellipodia by nucleation of actin filament must be coordinated to induce directional cell motility^47^. On the one hand, WAVE protein may be an essential regulator of lamellipodia formation with Arp2/3 complex, to initiate new filaments as a branch on the side of an existing filament^48–50^. On the other hand and more generally, extension of a single lamellipodium can be regulated by several other proteins^47,51^. Among them, the small Rho-GTPases were recognized as controlling the formation of actin cytoskeleton and cell morphology; Rac generates protrusive forces through actin polymerization in F-actin at the leading edge, Cdc42 proteins have a crucial role at the front of cells to control direction of migration and Rho is responsible to rear tail retraction by stress fibers formation^52,53^. Taken together, these molecular mechanisms generate a front-rear axis essential for unilateral cell polarity^51,53–55^. Indeed, Dua *et al*., confirmed the importance of RhoA and Rac1 using a Pentoxifylline molecule which was able to avoid migration of B16F10 cells, affecting expression of these small molecule reducing stress fibers and lamellipodial protrusions^56^. Similarly, another study also demonstrated the importance of cholesterol in membrane-cytoskeletal dynamic, since cholesterol depletion results in Src kinase-mediated Rho activation and stress fibers formation^57^. Hence, being correlated to these releases our data suggested a malfunction of Rho-GTPases signaling pathways. Moreover, as actin cytoskeleton in connection with the plasma membrane provided the structure and the shape of cells, the cellular disorientation and disruption of actin network observed under *Phenacetinum* 4CH treatment could be closely related.

Finally, the cascade of these multiple events under homeopathic drug treatment, leads to decrease the B16 cells velocity leading to a reduction of the distance traveled by B16 dispersed cells, after 2 h and in a sustainable way up to 24 h. However, during tumor progression, it is the development of aberrant and individual cell migration which promotes metastatic dissemination to a site or organ distant from a primary tumor^1,58^. In fact, many reports have described that effective mesenchymal cell migration was composed through a cyclic persistent process of different steps: protrusion, adhesion, contraction and retraction of cells towards a specific direction^2,3,59^. In conclusion, our results propose that *Phenacetinum* 4CH influences protrusion and retraction processes, limiting the capacity of single cell to cover local distance.

Taken together, our data allowed us to highlight an *in vitro* action mechanism of *Phenacetinum* 4CH homeopathic dilution, as a specific disruptor of murine melanoma cell migration on a fibronectin matrix. Indeed, the likely insertion of *Phenacetinum* 4CH into the plasma membrane of B16 cells, strongly comforted by MD simulations, appears to disturb their organization as evidenced by an increase of Lo phases. Also, we suggest that these molecular phenomena will impair signaling pathways from lipid rafts and cholesterol contained in the plasma membrane, involve probably different second messengers (such as PIP_2_, Rho-GTPases or Ca^2+^) which may affect the actin network and result in the loss of directional cell migration. The impact of *Phenacetinum* 4CH on the signaling platforms and the disturbing underlying cascades remains to be studied. However, this pioneering work properly addresses the effect of homeopathy on tumor progression cells lines, as a way of associating it to conventional subsequent treatments.

## Methods

### Cells lines and reagents/antibodies

B16F1, B16F10 (murine melanoma cell lines), NIH3T3 (murine embryo fibroblast cell line) and MEF (murine embryonic fibroblast) were purchased from ATCC. The cells were cultured in RPMI 1640 supplemented with 10% fetal bovine serum (FBS) in standard conditions (5% CO_2_, 37 °C). Both cell lines were used at low passage number (<15) and were mycoplasma free (MycoAlert; Lonza). Fibronectin, Mitomycin C and Laurdan were obtained from Sigma Aldrich (France). RPMI 1640 medium was obtained from Gibco (France). Fetal Bovine Serum (FBS) was obtained from ATCC. The probe used in this study is an anti-phalloïdin coupled to Alexa-fluor 488 (Invitrogen). Homeopathic dilution, *Phenacetinum* 4CH, was obtained from BOIRON laboratories (Messimy, France): 1 g of Phenacetine was firstly diluted in 99 ml d′H_2_O. This dilution was repeated four times (1 × 10^−8^) to obtain a final concentration at 0.563 nM.

### Cell viability assay

The cells were seeded at a density of 5 × 10^3^ cells/well in a 96-well culture plate. Homeopathic drugs are administrated in wells for 5% and stopped after 0 h or 24 h of incubation (5% CO_2_, 37 °C). For that, 20 *μ*L of MTT solution at [5 mg/ml] were added in wells and incubated during 3 h (5% CO_2_, 37 °C). The media were gently removed and 100 *μ*L of DMSO were added to dissolve formazan crystals. MTT reduction was quantified by measuring the light absorbance at 570 nm using the reader microplate (TECAN, Infinite). Each experiment was repeated three times.

### Migration into dispersed cells assay

The B16 cells were seeded at a density of 7.5 × 10^3^ cells/well and the MEF cells at 3.5 × 10^3^ cells/well in a 24-well culture plate. To put it briefly, cells were grown on a fibronectin-coated surface (7 *μ*g/mL) for 24 h, then treated with mitomycin C (1 *μ*g/mL) for 2 h. Then after, cells were rinsed twice with medium low serum and treated with 5% of homeopathic dilution in RPMI 1640 0.5% FBS. The plate was placed on the stage of a fully-motorized inverted microscope (AxioObserver Z1, Zeiss) equipped with an environmental chamber (5% CO_2_, 37 °C). For each experiment, time-lapse images of several fields were acquired every 10 min over a 24 h period, using a 10x phase contrast objective (Numerical Aperture N.A. 0.3, 0.65 μm/pixel, binning 1). Cell tracking was performed using *Manual Tracking* plugin of Fiji where 60 individual cells were tracked per treatment for each experiment during 24 h of the migration. The data obtained were used with Chemotaxis software tool for another representation of tracks. At least three experiments were conducted for each condition examined in the present study.

### Transwell migration assay

A 24-well Transwell chamber (Greiner Bio-One, Dutscher, France) with a 8-*μ*m pore PET membrane, was used to perform the migration assay and coated with fibronectin at 7 *μ*g/mL. The lower chamber was filled with 600 *μ*L RPMI 1640 conditioned medium (made with NIH3T3 cell line). Then, 200 *μ*L B16F1 or B16F10 melanoma cells suspension (2.5 × 10^5^ cells/mL with 0.5% FBS), containing 5% of homeopathic dilution, were added to the insert. The cells were allowed to migrate at 37 °C with 5% CO_2_ over 6 h. The inserts were washed in PBS and fixed with methanol for 15 min. Non-migrating cells were removed from the upper surface of the inserts by gently scrubbing with a cotton-tipped swab. Each PET membrane were cut and stained with mounting medium DAPI (ProLong Gold DAPI, ThermoFischer), between blades and slats. For counting, 10 pictures were taken per membrane, and each condition was made in duplicate. Experiments were performed at least in triplicate.

### Cell circularity and cell polarity

Cell morphology was quantified using circularity index on ImageJ software by the following formula: 4π(area)/(perimeter)^2^. This formula gives a circularity index ranging from 0 at 1, where value 0 corresponding to elongated shape and value 1 to a rounded morphology. Cells were chosen randomly from tracking obtained previously by time-lapse movies, and the average of the contours was made on 0, 6, 12, 18 and 24 h. Experiments were performed at least in triplicate.

### Atomic force microscopy

The AFM utilized in this study is the Bioscope CatalystTM (Bruker, Billerica, USA) coupled to a Nikon Eclipse Ti inverted microscope (Nikon, Tokyo, Japan). The glass-bottom petri dishes (50 mm in diameter, WillCo Wells, Amsterdam, the Netherlands) were put onto the AFM stage and observed with Bright Field illumination in order to locate the cells. One day prior to the experiments, B16 cells were seeded at a density of 5 × 10^4^ cells/mL and MEF cells at 2.5 × 10^4^ cells/mL on the plates previously coated with fibronectin (7 μg/ml). After a 24 h period, the cells were rinsed twice with medium low serum and treated with 5% of homeopathic dilution in RPMI 0.5% FBS, for 1 h at 37 °C, 5% CO_2_. All images were captured in Peak Force Quantitative Nanomechanical Mapping (PFQNM) mode. PFQNM-LC-A-CAL probes, having a nominal spring constant of 0.1 N/m and a resonance frequency of ~45 kHz were used to image the cells, the tips being calibrated with the company protocol and as previously described where the Young’s moduli were calculated by using a Sneddon fit^60^. For PFQNM experiments, we used a PeakForce frequency of 0.25 kHz in order to maximize the contact time between the tip and the sample. The PeakForce amplitude was set at 1 μm. The loading force was lowered down to a few tens of pN to avoid generating mechanical stress of the cells. The indentation force applied on cells was between 50 and 100 nm, directly on cortical actin. Images were captured in culture medium at a resolution of 256 or 128 pixels per line, at 37 °C using a Perfusing Stage Incubator. Regarding the Young’s modulus calculation, a minimum of 3 analysis on 3 different cells (perinucleus areas were avoided) were performed and the experiments were triplicated for each sample type.

### Immunofluorescence staining

B16F1 and B16F10 were plated at low density onto plastic LabTek (Nunc, Dutscher, France) previously coated with 7 *μ*g/mL of fibronectin for 12 h at 4 °C and blocked with 1% BSA. Cells were incubated on the LabTek for 24 h at 37 °C in 5% CO_2_ to allow spreading. Subsequently, cells were treated with 5% homeopathic dilution in medium low serum during 1 h and 6 h at 37 °C and 5% CO_2_. They were rinsed once gently with medium low serum at 37 °C and once with glutaraldehyde 0.1% in PBS. Cells were fixed with glutaraldehyde 0.5% for 10 min at room temperature (RT). After two rinses with PBS, cells were saturated with 10% BSA for 1 h at RT. Cells were incubated with Alexa Fluor^TM^ 488 Phalloidin probe for 1 h, in 2% BSA/TBS-Triton X100 (1:100 dilution). After incubation, LabTek were washed in 0.1% Triton/TBS and mounted onto slides using mounting medium DAPI. Observation of LabTek were taken at fluorescent microscope (BX51WI, Olympus) Ex/Em = 493/517 nm.

### Laurdan two-photon microscopy

Laurdan is an amphiphilic fluorescent probe able to penetrate a biological membrane, and to detect changes in membrane phase properties through its emission spectral shift. B16F1 and B16F10 were plated at low density onto a cell culture dish (35 mm diameter, FluoroDish WPI), previously coated with 7 *μ*g/mL fibronectin for 12 h at 4 °C and blocked with 1% BSA. At the end of the day, cells were rinsed twice with medium low serum, then 1.9 ml of this medium with 2 μL of Laurdan (2-dimethylamino(6-lauroyl)naphthalene) at [5 μM] were added overnight at 37 °C. In the following morning, 100 μL of drug was added directly into a well, and around 10 cells were imaged with confocal microscope (LSM 710 NLO ZEISS). Laurdan intensity images were recorded simultaneously with emission in the range of 400–460 nm and 470–530 nm. Membrane fluidity was measured in terms of ratio of emission intensities by using Generalized Polarization (GP) value^61^, defined as

$$GP=\frac{I(400-600)-GI(470-530)}{I(400-460)-GI(470-530)}$$

### Molecular dynamic simulations

Phenacetin was studied by means of MD simulations in the presence of different compositions of membranes as constituted of 256 POPC, of 256 DPPC and finally of DPPC:cholesterol (230:26). All simulations were performed using the CHARMM36 force field^62^ and the phenacetin topology was generated with the CHARMM general force fields (Program version 1.0.0, CGenFF version 3.0.1)^63^. Membrane systems were generated by using the CHARMM-GUI membrane builder^64–67^ and the box filled with TIP3P water^64^. Either one or 25 phenacetines were placed 1 nm away from the membrane surface. All the systems studied were equilibrated by using the step equilibration proposed by the CHARMM-GUI membrane builder; a minimization by steepest descent of 1,000 steps, two NVT and four NPT simulations with increasing length and time step and at the end decreasing restraints force constant on lipids phosphate positions and dihedral angles. Phenacetines were also kept under position restraints before production simulations were performed.

### Statistical analysis

All data were expressed as mean ± SEM of at least three independent experiments. Software GraphPad Prism 6.0 was used for statistical analysis. The significance of the differences in the samples was measured by One Way ANOVA with Dunnett t-test. Differences were considered significant at p < 0.05.

## Supplementary information

S1 S2 S3 S4 S5
