# Supplementary material for: Low-diluted Phenacetinum disrupted the melanoma cancer cell migration
Source: Sci Rep. 2019 Jun 24;9:9109. doi: 10.1038/s41598-019-45578-1 (PMC6591484; doi:10.1038/s41598-019-45578-1)
Supplement: Supplementary file 1 — S1 S2 S3 S4 S5 [file 41598_2019_45578_MOESM1_ESM.pdf]

Low-diluted *Phenacetinum* disrupted the melanoma cancer cell migration

Camille Fuselier[1], Christine Terryn[2], Alexandre Berquand[3], Jean-Marc Crowet[1], Arnaud Bonnomet[2], Michael Molinari[3], Manuel Dauchez[1], Laurent Martiny[1] and Christophe Schneider[1*]

[1] CNRS UMR7369 MEDyC, University of Reims Champagne-Ardenne, Reims, France

[2] Plateform PICT, University of Reims Champagne-Ardenne, Reims, France

[3] LRN EA 4682, University of Reims Champagne-Ardenne, Reims, France

* corresponding author: christophe.schneider@univ-reims.fr

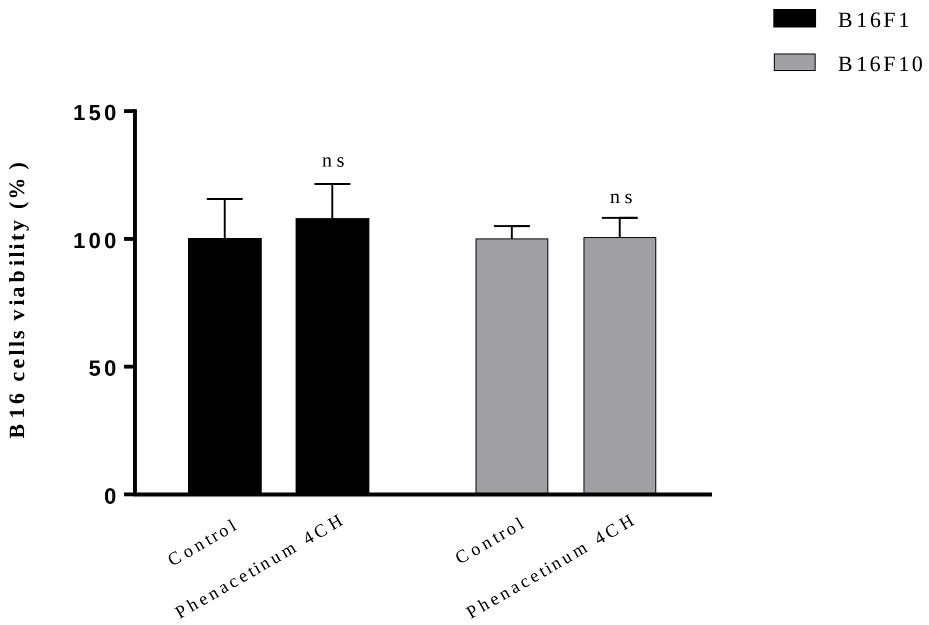

Figure S1. Influence of *Phenacetinum* 4CH on B16F1 and B161F0 cell viability. Cell viability was studied by MTT [1mg/ml] colorimetric test after 24h of treatement with 5% of *Phenacetinum* 4CH for B16F1 cells (A) and B16F10 cells (B). Data are expressed as means ± SEM (n=3, ns=no significant, student t test).

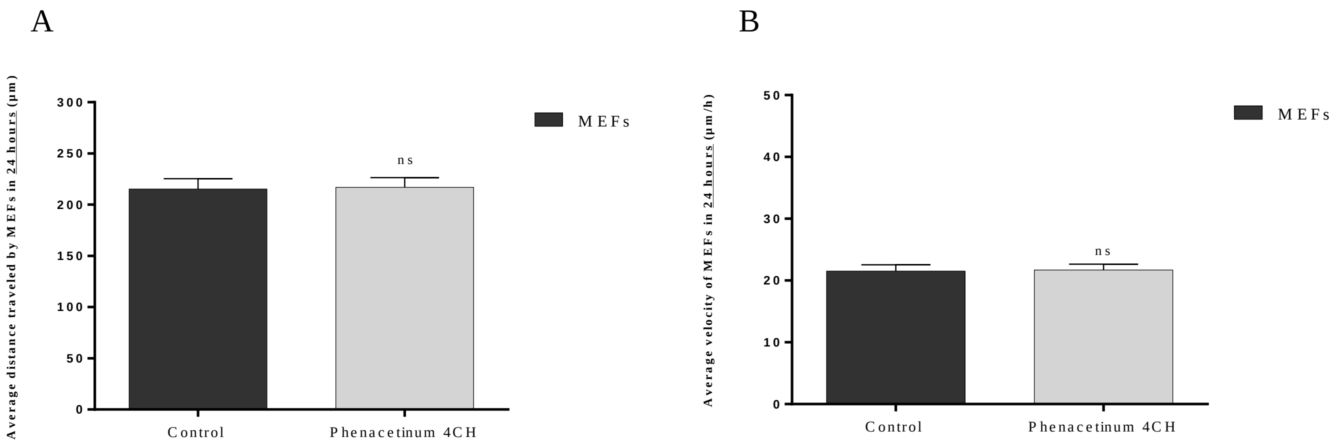

Figure S2. Influence of *Phenacetinum* 4CH on MEFs cell dispersed migration. Cell tracking using *Manual Tracking* plugin of Fiji with 20 individual cells treated for each experiment during 24 h of the migration. Traveled distances (A) and velocity (B) of migration are analyzed. Datas are expressed as means ± SEM (n=3, ns=no significant, student t test).

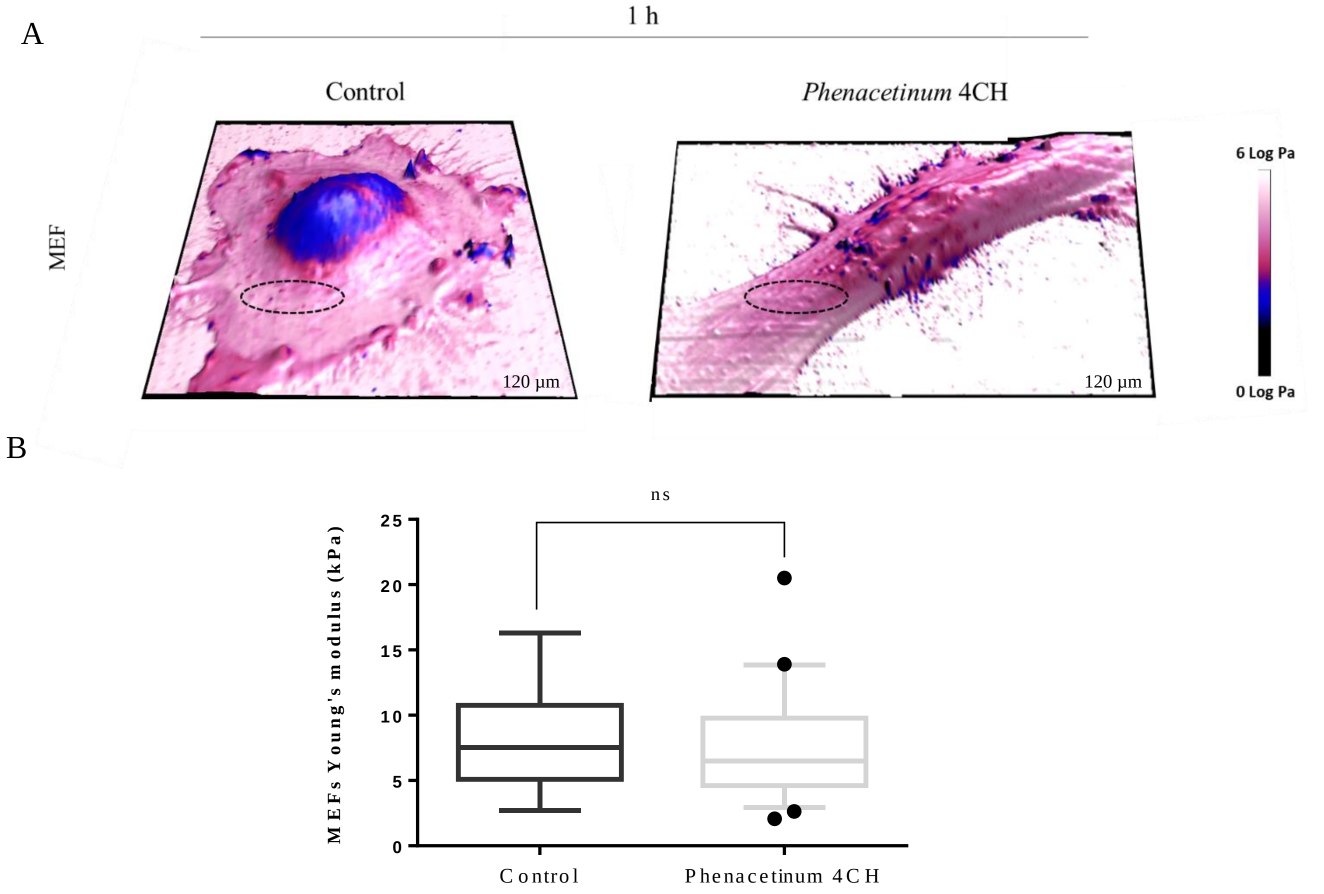

Figure S3. Influence of *Phenacetinum* 4CH on MEFs cell stiffness. Cell stiffness was analyzed by AFM, and images were captured in PFQNM mode. Regarding the Young's modulus calculation, a minimum of 3 analysis on 3 different cells (perinucleus areas were avoided, black dotted circle) were performed and the experiments were triplicated for each sample type/ Data are expressed as means ± SEM and represented in box and whiskers (n=3, ns=no significant, student t test).

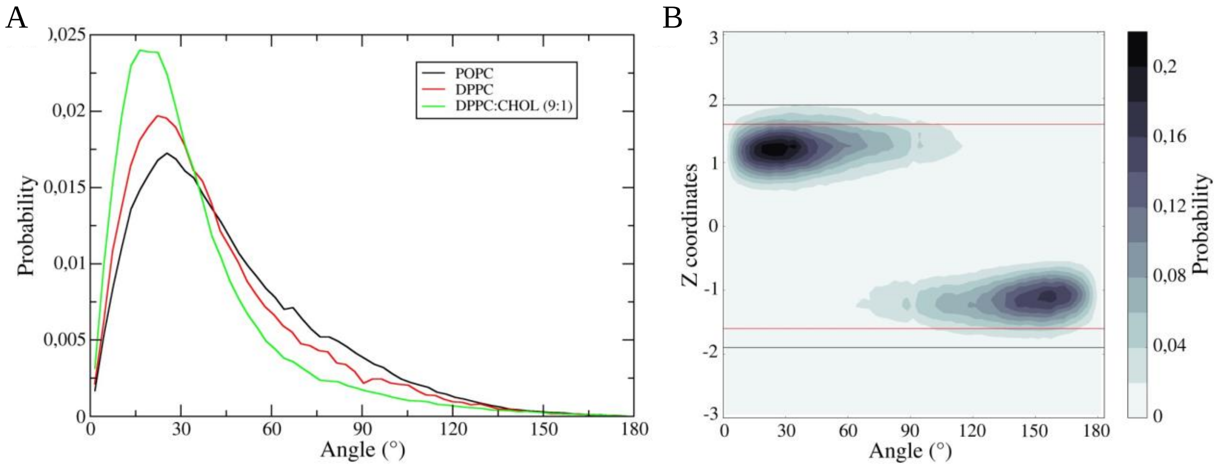

Figure S4. Positions of phenacetin in membranes. (A) Distribution of angles between the phenacetin long axis and the perpendicular axis related to the membrane. (B) Distribution of phenacetin in the POPC membrane according to the Z coordinate and the angles between the phenacetin long axis and the perpendicular axis. Black and red lines correspond to phosphate (1.9 nm) and glycerol (1.6 nm) positions.

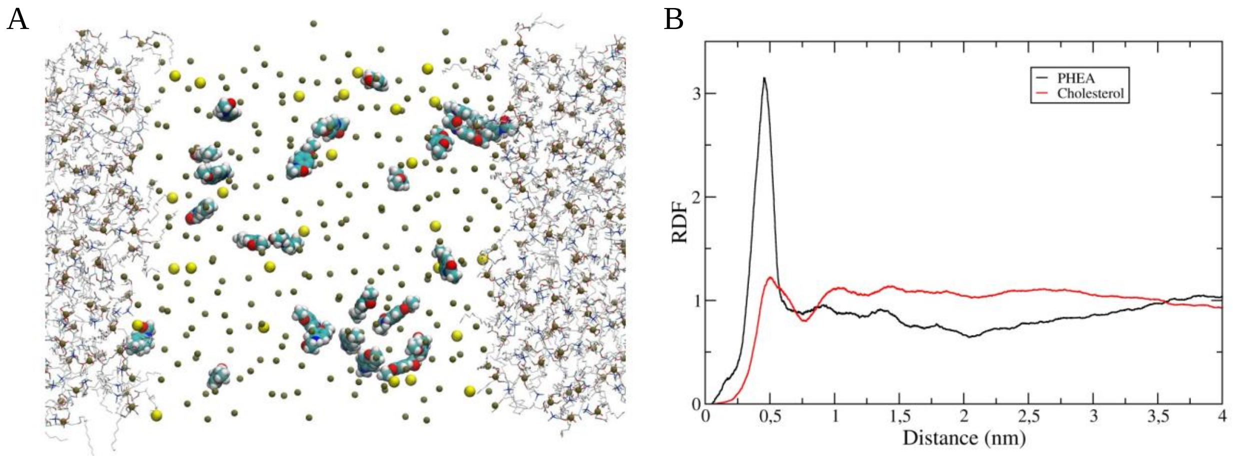

Figure S5. Molecular dynamics simulations of 25 phenacetins in presence of a POPC:CHOL bilayer. (A) Top view of the system with phosphorus (tin color), cholesterol oxygen (yellow) and phenacetins depicted using the van der Waals representation and the lipids with a line representation. (B) Radial distribution function (RDF) between phenacetins and between phenacetins and cholesterol.
